# SARS Risk Perceptions in Healthcare Workers, Japan

**DOI:** 10.3201/eid1103.040631

**Published:** 2005-03

**Authors:** Teppei Imai, Ken Takahashi, Tsutomu Hoshuyama, Naoki Hasegawa, Meng-Kin Lim, David Koh

**Affiliations:** * University of Occupational and Environmental Health, Kitakyushu, Japan;; † Keio University, Tokyo, Japan;; ‡ National University of Singapore, Singapore

**Keywords:** SARS, Occupational Health, Health Personnel, Infection Control, Questionnaires, Epidemiology

## Abstract

In coping with severe acute respiratory syndrome (SARS), infection control measures are a key aspect of protecting healthcare workers. We conducted a survey concerning perception of risk and countermeasures for SARS in 7 tertiary hospitals in Japan from July through September 2003, immediately after the SARS epidemic in neighboring countries. Based on 7,282 respondents out of 9,978 questionnaires administered, we found the perception of risk to be relatively high and the perception of countermeasures at the institutional level to be relatively low. Knowledge of preventive measures, concept of (opinions regarding) institutional measures, and perception of risk differed substantially among the 3 job categories, notably between physicians and nurses. The concept of institutional measures was the most important predictor of individual perception of risk. In view of the potential for future epidemics, planning and implementing institutional measures should be given a high priority.

Severe acute respiratory syndrome (SARS) has been reported in 30 countries with a total of 8,096 probable cases and 774 deaths as of July 31, 2003. A large proportion of these SARS outbreaks occurred in hospitals, and 21% of probable SARS cases involved healthcare workers (1). Protecting healthcare workers is essential from the standpoints of both public and occupational health. Experience in hospitals has suggested that appropriate infection control measures, including use of personal protective equipment, personal hygiene, and environmental measures, such as area isolation, protect healthcare workers from SARS (2,3). During the SARS epidemic, hospitals in affected areas emphasized training and issued guidelines on infection control and use of personal protective equipment (4–6). To prepare for future potential outbreaks of SARS and other emerging infectious diseases, implementing appropriate infection control measures in healthcare settings and assessing the efficacy of those measures in the postepidemic period are necessary.

Japan was one of the few Asian countries to be spared from the SARS epidemic in 2003. Although Japan did not experience cases of SARS, healthcare workers in Japan likely felt insecure in their work environment because of the situation in neighboring countries. Quah et al. (7) reported that the anxiety level of the general population in Singapore was low at the height of the SARS epidemic (55% of the respondents reported a low anxiety level). In contrast, Nickell et al. (8) reported that, in a teaching hospital in Toronto, the SARS outbreak had substantial psychological effects on healthcare workers, whose General Health Questionnaire scores suggested that "a probable case of emotional distress" was more than double the level of the general population. However, the level of anxiety (i.e., perception of risk) among healthcare workers has yet to be evaluated in Japan. Infection control measures and other administrative support also must be examined at the institutional level, which may influence the perception of risk among healthcare workers.

Another point of interest is the comparison between the overall preparedness of Japan for SARS and the preparedness of other countries. Thus, we joined an international collaborative effort to study the perception of risk and countermeasures for SARS among healthcare workers and conducted a survey concerning those issues among healthcare workers in Japan. The objective of the present analysis was 2-fold: 1) to assess healthcare workers' perception of risk, knowledge of preventive measures, and perception of infection control measures at the institutional level and 2) to evaluate the interrelationships among these factors, with a focus on institutional measures.

## Materials and Methods

### Study Population

The study population comprised 9,978 healthcare workers working at 7 tertiary-level hospitals distributed throughout Japan; 4 of the hospitals are university-affiliated, 2 are municipal, and 1 is private. The study participants held a wide range of jobs in each institution. The questionnaire was administered from July through September 2003. Overall, 7,463 healthcare workers responded to the questionnaire (crude response rate 74.8%). After missing or invalid responses for sex, age, or job category were excluded, 7,282 were finally analyzed (valid response rate 73.0%) (Table 1).

**Table 1:** Demographic characteristics of respondents*

Variable	n (%)
Age, y (mean 35.6 ± SD 11.2)	
<35	3,963 (54.4)
>35	3,319 (45.6)
Sex	
Women	5,077 (69.7)
Men	2,205 (30.3)
Job category	
Physicians	1,370 (18.8)
Nurses	3,274 (45.0)
Others†	2,638 (36.2)
Tenure at this job, y (mean 11.1 ± SD 9.6)	
<10	3,884 (54.1)
>10	3,292 (45.9)
Type of facility	
University hospitals (4 facilities)	5,163 (70.9)
Municipal hospitals (2 facilities)	1,344 (18.5)
Private hospital (1 facility)	775 (10.6)
Total	7,282 (100.0)

*SD, standard deviation. †Others include nursing assistant, social worker, pharmacist, clinical and radiologic technologist, physical therapist, occupational therapist, speech therapist, managerial staff, clerk, educational and research staff, building maintenance staff, cleaner, nutritionist, and licensed cook.

### Questionnaire

This study formed part of an international collaborative study involving healthcare workers in Singapore, China, Taiwan, Canada (Toronto), and Japan. The questionnaire was developed in English at the National University of Singapore, translated into Japanese, and adapted to accommodate background conditions (i.e., no outbreak). The questionnaire was anonymous, and procedures involving human participants were approved by the institutional review board of the University of Occupational and Environmental Health, Japan.

The questionnaire included 24 items regarding knowledge of preventive measures (15 items), concept of (opinion regarding) institutional measures (4 items), and perception of risk (5 items) (Appendix). These 24 items were measured on a 7-point scale for responses (strongly agree, agree, probably agree, probably disagree, disagree, strongly disagree, and not applicable). In the statistical analyses, we dichotomized this scale into positive response (strongly agree, agree, and probably agree) and negative response (strongly disagree, disagree, and probably disagree) after excluding "not applicable."

To assess knowledge of preventive measures, we analyzed responses to questions regarding the effectiveness of measures to avoid contracting SARS (personal protective equipment, personal hygiene, environmental measures). The 15 items are shown in Appendix. The correct response to each item was designated on the basis of World Health Organization (WHO) guidelines (2) and other findings. The correct responses for the 15 items were a positive response for all items except "paper mask" and "gauze mask," which required a negative response. To assess concept (opinion) of institutional measures, we used 4 items regarding "clear policies and protocols," "specialist available," "adequate training," and "effectiveness." To assess perception of risk, we used 5 items regarding "avoidance of patient," "acceptance of risk," "little personal control," "fear," and "job change," as indicators (Appendix).

We quantified the degree of concept of institutional measures and that of knowledge of preventive measures by calculating the institutional (I) and knowledge (K) scores. The I-score was defined as the total number of positive answers to the 3 specific questions regarding "clear policies and protocols," "specialist available," and "adequate training"; the maximum possible I-score was 3 points. "Effectiveness" was excluded from the calculation of the I-score because it could be looked upon as a combined, general concept of institutional measures. The K-score was defined as the total number of correct (either positive or negative) answers to the 15 questions regarding the knowledge of preventive measures; thus the maximum possible K-score was 15 points. The K-score was categorized as high (11–15 points), middle (6–10 points), or low (0–5 points). Cronbach's α was 0.87 for the K-score and 0.76 for the I-score, which indicated a high degree of internal consistency for each score.

### Statistical Analysis

The chi-square test was used to evaluate differences in the proportion of respondents according to job category (physician, nurse, and other), sex, age, and type of facilities. The Student *t* test was used to evaluate differences in the mean value between 2 groups, and analysis of variance was used to evaluate differences in the mean value among 3 groups. Logistic regression analyses were used to identify factors associated with the overall concept of the effectiveness of institutional measures ("effectiveness") and perceptions of risk ("avoidance of patient" and "acceptance of risk") as the dependent variables. The independent variables were the I-score (0, 1, 2, and 3 points), the K-score (low, middle, and high), age (<35 years old, >35 years old), sex (men, women), fear (–, +), and type of facility (nonuniversity hospital, university hospital). The logistic regression model was applied to each of the 3 job categories and to all participants. Spearman's correlation coefficients among 6 independent variables were <0.26, and no strong correlations were seen among them. Data were analyzed by using SPSS, version 11.5J (SPSS Inc., Chicago, IL, USA) for Windows and SAS V8 (SAS Institute Inc., Cary, NC, USA). All reported p values are 2-tailed, and p < 0.05 was considered statistically significant.

## Results

Levels of knowledge of preventive measures, concept of institutional measures, and perception of risk are shown in Table 2 (see Table A1 for complete data). The proportion, mean score, or both were calculated for each item or category according to job category (physician, nurse, and other), sex, age, and type of facility, as well as the total. As shown in Table 3, the distribution of job categories was significantly different between the 2 types of facility (university hospitals and nonuniversity hospitals), with a higher proportion of physicians and lower proportion of nurses in university hospitals. The corresponding proportion did not differ substantially between municipal and private hospitals, so we categorized the 2 types as nonuniversity hospital for further analyses.

**Table 2:** Knowledge of preventive measures, concept of institutional measures, and perception of risk by job category, sex, age, and type of facility*

Questionnaire item	Job category			Sex		Age		Type of facility		
	Physicians	Nurses	Other	Men	Women	<35 y	>35 y	University	Nonuniversity†	Total
Knowledge of preventive measures										
Area isolation	96.0	99.3	97.8	96.3	98.9	98.1	98.2	97.8	98.9	98.1
Hand washing	95.6	99.2	97.8	96.0	98.9	98.1	97.9	97.8	98.7	98.0
Alcohol rubs	87.0	94.6	95.1	89.8	94.9	93.2	93.5	93.2	93.6	93.3
Prominent notices	86.8	91.2	89.9	86.1	91.6	89.7	90.2	89.5	91.0	89.9
N95 mask	86.2	89.5	83.8	85.7	87.5	86.4	87.6	85.6	90.1	86.9
Gloves	73.7	82.6	78.2	74.8	81.3	78.0	81.0	77.3	84.3	79.3
Gowns	63.5	74.5	58.8	62.0	69.1	65.8	68.5	63.9	74.4	67.0
Surgical mask	64.5	62.5	66.4	63.6	64.5	64.6	63.8	63.8	65.1	64.2
Temperature checks	51.2	61.5	65.4	58.1	62.2	61.6	60.1	60.5	61.9	60.9
Hair cover	55.1	63.9	56.6	56.3	61.1	56.9	63.1	56.1	68.3	59.7
Paper mask	64.3	62.3	51.6	61.5	58.0	56.9	61.8	59.5	57.9	59.0
Goggles	57.7	56.3	55.3	56.7	56.0	51.9	61.7	52.9	64.2	56.2
Gauze mask	58.5	58.6	46.7	54.4	54.5	51.5	58.3	54.3	54.8	54.5
Shoe cover	50.6	55.5	51.9	50.7	54.4	50.7	56.5	49.4	62.6	53.3
Limiting visitors	29.9	41.3	30.5	31.6	36.9	33.4	37.6	32.6	41.9	35.3
Concept of institutional measures										
Clear policies and protocols	62.8	70.6	59.4	62.6	66.4	61.6	69.7	62.8	71.2	65.2
Specialist available	42.6	59.6	50.4	45.4	56.5	48.8	58.3	49.0	62.8	53.0
Adequate training	29.4	48.9	31.9	31.7	42.4	35.2	43.8	35.7	47.1	39.1
Effectiveness	27.2	34.0	29.5	30.6	31.7	28.1	34.6	28.4	37.6	31.1
Perception of risk										
Avoidance of patient	86.9	93.4	92.1	87.3	93.7	93.1	90.0	91.1	93.3	91.7
Acceptance of risk	69.5	64.7	60.9	67.0	63.1	62.3	66.6	64.0	64.8	64.3
Little personal control	59.8	61.7	59.8	59.2	61.3	60.6	60.7	60.5	61.1	60.6
Fear	48.9	60.6	52.1	48.7	58.2	55.8	54.7	52.2	62.9	55.3
Job change	14.3	34.1	24.7	15.7	31.9	30.7	22.5	24.1	33.8	27.0

*Data are presented as percentages, number of positive responses divided by number of respondents answering each question (except for paper mask and gauze mask, where negative responses were counted). Positive responses include "probably agree," "agree," and "strongly agree"; negative responses are "probably disagree," "disagree," and strongly disagree." Detailed information, including n's, distribution of scores, and p values (based on chi-square test for difference in proportion, *t* test for difference in 2 means, and analysis of variance [ANOVA] for differences in 3 means), is available in the full table in Table A1. †Nonuniversity includes municipal hospitals (2 facilities) and private hospitals (1 facility).

**Table 3:** Job categories by type of facility

Type of facility	Physicians, n (%)	Nurses, n (%)	Others, n (%)	p value*	Total
University hospital (4 facilities)	1,116 (21.6)	2,225 (43.1)	1,822 (35.3)	<0.001	5,163 (100.0)
Nonuniversity hospital (3 facilities)	254 (12.0)	1,049 (49.5)	816 (38.5)		2,119 (100.0)
Municipal hospital (2 facilities)	153 (11.4)	688 (51.2)	503 (37.4)		1,344 (100.0)
Private hospital (1 facility)	101 (13.0)	361 (46.6)	313 (40.4)		775 (100.0)
*p value based on chi-square test between university and nonuniversity hospitals.					

For knowledge of preventive measures, the overall correct response rates were, in descending order, area isolation (98.1%), hand washing (98.0%), alcohol rubs (93.3%), prominent notices (89.9%), N95 mask (86.9%), gloves (79.3%), gowns (67.0%), surgical masks (64.2%), temperature checks (60.9%), hair cover (59.7%), paper mask (59.0%), goggles (56.2%), gauze mask (54.5%), shoe cover (53.3%), and limiting visitors (35.3%). The correct response rate differed significantly among job categories for all items except for goggles. As a general trend, physicians ranked third for 9 items, nurses ranked first for 10 items, and others ranked second for 7 items. The K-score distribution and mean indicated the highest score for nurses, intermediate for physicians, and lowest for others. The correct response rate differed significantly between men and women for all items except goggles, gauze mask, and surgical mask. As a general trend, women ranked higher than men for 13 out of 15 items. Accordingly, the K-score distribution and mean indicated a significantly higher score for women. This trend was observed in physicians but not in nurses when the analysis was conducted separately for each group (data not shown). The correct response rate differed significantly between the 2 age categories for 8 items. As a general trend, older workers (>35 years old) ranked higher for 12 of the 15 items. However, neither the K-score distribution nor the mean K-score indicated a higher score for older workers. The correct response rate differed significantly between the 2 types of facilities for 9 items. As a general trend, nonuniversity hospital ranked higher for 14 of the 15 items. Accordingly, the K-score distribution and mean indicated a significantly higher score for nonuniversity hospital.

For concept of institutional measures, the overall proportion of positive responses were, in descending order, clear policies and protocols (65.2%), specialist available (53.0%), adequate training (39.1%) (concept of respective institutional measures), and effectiveness (31.1%) (overall concept of effectiveness of institutional measures). For all items, the positive response rate differed significantly among the 3 job categories, with nurses consistently ranked the highest. The I-score distribution and mean indicated the highest score for nurses, intermediate for physicians, and lowest for others. For all items except for the overall concept of effectiveness, the rate of positive responses was significantly higher for women than men. The I-score distribution and mean indicated a higher score for women than men. For all items, the positive response rate was significantly higher for older workers than younger workers. Accordingly, the I-score distribution and mean indicated a significantly higher score for older workers than younger workers. For all items, the positive response rate was significantly higher for nonuniversity hospital than university hospital. Accordingly, the I-score distribution and mean indicated a significantly higher score for nonuniversity hospital than university hospital.

For perception of risk, the overall positive response rates were, in descending order, avoidance of patient (91.7%), acceptance of risk (64.3%), little personal control (60.6%), fear (55.3%), and job change (27.0%). The positive response rate differed significantly among the job categories for all items except little personal control. Nurses ranked highest for avoidance of patient (93.4%), whereas physicians ranked highest for acceptance of risk (69.5%). Nurses showed the highest level of fear (60.6%) and physicians the lowest (48.9%). Nurses had the highest tendency to consider job change (34.1%) and physicians the lowest (14.3%). The positive response rate differed significantly between men and women for all items except little personal control. Compared to men, women had a significantly higher proportion of positive responses to avoidance of patient (93.7% vs. 87.3%, p < 0.001), fear (58.2% vs. 48.7%, p < 0.001), and job change (31.9% vs. 15.7%, p < 0.001) and lower proportion of positive responses to acceptance of risk (63.1% vs. 67.0%, p = 0.002). The positive response rate differed significantly between the 2 age categories for all items except little personal control and fear. Compared to younger workers, older workers had a lower proportion of positive responses to avoidance of patient (90.0% vs. 93.1%, p < 0.001) and job change (22.5% vs. 30.7%, p < 0.001), and a higher proportion of positive responses to acceptance of risk (66.6% vs. 62.3%, p < 0.001). The positive response rate differed significantly between university hospital and nonuniversity hospital for all items except acceptance of risk and little personal control. Compared to university hospitals, nonuniversity hospitals had a significantly higher proportion of positive responses to avoidance of patient (93.3% vs. 91.1%, p = 0.002), fear (62.9% vs. 52.2%, p < 0.001), and job change (33.8% vs. 24.1%, p < 0.001).

As shown in Table 4, logistic regression analyses indicated that effectiveness (as overall conception of effectiveness of institutional measures) was positively associated with the I-score in all 3 job categories and with age in 1 job category (others). Effectiveness was negatively associated with fear in 2 job categories (physicians and others) and with type of facility in 2 job categories (nurses and others). Avoidance of patient was positively associated with fear in all 3 job categories, with gender in 1 job category (others), and with K-score in 1 job category (physicians). Avoidance was negatively associated with I-score in all 3 job categories and with age in 1 job category (nurses). Acceptance of risk was positively associated with I-score in all 3 job categories, with age in 1 job category (nurse), and fear in 1 job category (others). Hence, the I-score was a significant positive predictor of effectiveness (as overall conception of effectiveness of institutional measures) in all 3 job categories, a significant negative predictor of avoidance of patient in 2 job categories (physician and others), and a significant positive predictor of acceptance of risk in all 3 job categories.

**Table 4:** Factors associated with concept of institutional measures (effectiveness) and perception of risk (selected factors) by job category

Variable*	OR (95% CI)†			
	Physician (N = 1,370)	Nurse (N = 3,274)	Other (N = 2,638)	Total (N = 7,282)
Effectiveness				
I-score (0,1,2,3)	1.87 (1.65–2.13)	1.83 (1.69–1.98)	2.02 (1.84–2.22)	1.90 (1.80–2.01)
Age (>35 year)	1.23 (0.93–1.62)	1.19 (0.99–1.41)	1.42 (1.15–1.74)	1.25 (1.11–1.41)
K-score (low, middle, high)	0.89 (0.72–1.10)	1.01 (0.88–1.17)	0.96 (0.82–1.11)	0.97 (0.88–1.06)
Type of facility (university)	0.82 (0.59–1.15)	0.71 (0.60–0.84)	0.75 (0.60–0.92)	0.74 (0.65–0.83)
Sex (women)	0.79 (0.53–1.18)	0.63 (0.38–1.06)	0.97 (0.79–1.18)	1.00 (0.88–1.14)
Fear (+)	0.61 (0.46–0.80)	0.90 (0.76–1.06)	0.58 (0.47–0.70)	0.72 (0.64–0.81)
Avoidance of patient				
Fear (+)	1.91 (1.33–2.76)	2.56 (1.89–3.48)	2.32 (1.66–3.24)	2.31 (1.91–2.80)
K-score (low, middle, high)	1.42 (1.10–1.84)	1.01 (0.78–1.30)	1.23 (0.98–1.56)	1.19 (1.03–1.37)
Sex (women)	1.27 (0.76–2.13)	1.95 (0.91–4.21)	2.05 (1.50–2.82)	1.93 (1.59–2.33)
Age (>35 year)	1.00 (0.70–1.42)	0.58 (0.42–0.79)	0.84 (0.60–1.17)	0.81 (0.67–0.97)
Type of facility (university)	0.91 (0.57–1.43)	0.83 (0.59–1.16)	0.75 (0.52–1.08)	0.82 (0.66–1.01)
I-score (0,1,2,3)	0.80 (0.68–0.94)	0.87 (0.75–0.99)	0.80 (0.69–0.93)	0.81 (0.75–0.88)
Acceptance of risk				
I-score (0,1,2,3)	1.26 (1.11–1.42)	1.08 (1.01–1.16)	1.24 (1.14–1.35)	1.18 (1.12–1.24)
Age (>35 year)	1.05 (0.81–1.36)	1.27 (1.06–1.51)	1.11 (0.93–1.33)	1.10 (0.99–1.22)
Fear (+)	1.01 (0.78–1.30)	1.14 (0.97–1.33)	1.44 (1.21–1.71)	1.21 (1.09–1.34)
Sex (women)	1.01 (0.71–1.43)	0.65 (0.38–1.12)	0.85 (0.71–1.02)	0.82 (0.73–0.92)
K-score (low, middle, high)	0.90 (0.74–1.10)	1.05 (0.92–1.20)	1.04 (0.91–1.18)	1.03 (0.95–1.12)
Type of facility (university)	0.81 (0.58–1.14)	0.98 (0.83–1.15)	1.20 (0.99–1.45)	1.04 (0.93–1.17)

*Goodness-of-fit was satisfactory: ranged from [goodness-of-fit statistics = 0.49 with 8 df (p = 0.99)] for (effectiveness) x (physician) to [goodness-of-fit statistics = 12.71 with 8 df (p = 0.12)] for (nurse) x (avoidance), except for [goodness-of-fit statistics = 18.98 with 8 df (p = 0.02)] for (nurse) x (effectiveness) and [goodness-of-fit statistics = 18.38 with 8 df (p = 0.02)] for (others) x (effectiveness). †OR, odds ratio calculated by logistic regression; CI, confidence interval.

## Discussion

A substantial number of probable SARS cases were concentrated in Asian countries during the previous SARS epidemic (5,327 cases in China, 1,755 cases in Hong Kong, 346 cases in Taiwan, and 238 cases in Singapore as of July 31, 2003) (1). Accordingly, strict policies and administrative measures for infection control (e.g., mandatory quarantine and training of healthcare workers in infection control measures) were implemented in these countries (9–11). In contrast, no probable SARS cases were recorded in Japan, and thus administrative measures for infection control tended to be hypothetical (i.e., most countermeasures at the institutional level were voluntary) (12,13). As such, the Japanese situation is distinct from that in other Asian countries, and various aspects of knowledge, perception, and attitudes of healthcare workers regarding SARS are likely to differ between Japan and other Asian countries. To clarify this issue, we assessed the level of knowledge of preventive measures, concept of institutional measures, and perception of risk and their interrelationships in healthcare workers in Japan.

### SARS Knowledge, Concept of Institutional Measures, and Perception of Risk

Regarding knowledge of preventive measures, most respondents assigned relatively high importance to hand hygiene and area isolation but saw personal protective equipment as being of relatively low importance. This finding may be partly due to healthcare workers' not having previously used some of the protective equipment recommended for use with SARS patients (3). The use of personal protective equipment as countermeasures for SARS has been rightly advocated by various authors (10,14,15). Thus, adequately training healthcare workers in the use of personal protective equipment is an important aspect of reducing the incidence of SARS infection.

Regarding the concept of institutional measures, 40% of respondents believed that they had received adequate training; for example, less than half felt that they had adequate training in the use of masks. During the SARS epidemic, medical institutions were required by authorities to provide adequate training to healthcare workers in affected countries (5,6,10,11). Because no outbreaks were in Japan, however, Japanese institutions have not been forced to implement sufficient measures to adequately cope with future outbreaks of SARS and other emerging diseases.

Regarding perception of risk, although we did not compare healthcare workers with an external group, more than half (55%) of the healthcare workers surveyed indicated that they were afraid. Furthermore, a high proportion of healthcare workers preferred to avoid the patient (92%), although almost two thirds accepted the risk (64%). When these 2 items were cross-classified, 55% of respondents showed a mixed attitude (i.e., avoidance of patient [+] and acceptance of risk [+]), 32% showed a disloyal attitude (i.e., avoidance of patient [+] and acceptance of risk [–]), and 6% showed a loyal attitude (i.e., avoidance of patient [–] and acceptance of risk [+]). These results indicate a high level of fear and anxiety with complex psychology in Japanese healthcare workers, even in the absence of an epidemic.

Significant differences were seen in the level of knowledge and attitudes among the 3 job categories. Nurses showed the best knowledge of preventive measures and concept of institutional measures, while physicians showed the highest acceptance of risk. Both sex and job characteristics may have influences in this regard. Ninety-eight percent of nurses were women, whereas 84% of physicians were men. Quah et al. reported that, in Singapore, women showed better practice of SARS preventive measures than men among the general population (7). Similarly, our results indicated a higher level of knowledge regarding preventive measures for female physicians compared to male physicians. However, this trend was not observed within the nurse job category, although the number of male nurses was sufficiently small that separating the effect of sex was difficult. In terms of job characteristics, nurses may receive more official training in infection control than physicians, under the assumption that physicians are already knowledgeable. In fact, compared to physicians, nurses have higher levels of compliance with universal precautions (16) and hand-washing (17,18) in their respective countries. However, nurses tend to have higher job turnover rates than physicians, which reflect less stability or security in their profession. These factors directly and indirectly influence the response pattern among the 3 job categories.

### Interrelatedness of Knowledge, Concept of Institutional Measures, and Perception of Risk

In the logistic regression model, K-score, an indicator of knowledge of preventive measures, was not a significant predictor of perception of either risk or concept of institutional measures. This finding implies that professional knowledge has little, if anything, to do with positive perception of risk (in terms of accepting risk and not avoiding patients) and concept of institutional measures. However, the importance of providing accurate knowledge cannot be discounted solely on this ground. In contrast, I-score, an indicator of concept of institutional measures, was a significant positive predictor of concept of effectiveness and acceptance of risk and a significant negative predictor of avoidance of patient. In other words, a collective assertion of 3 specific institutional measures (clear policies and protocols, specialists available, and adequate training) had the greatest effect on a person's 2 different aspects of perception of risk and concept of the effectiveness of institutional measures. These findings corroborate earlier studies reporting that administrative support enhances compliance with universal precautions (19–21) and hand washing (17,18). Therefore, we infer that perception of institutional measures affects perception of risk and related behaviors.

Fear was a significant negative predictor of concept of effectiveness in 2 job categories (physicians and others) and a significant positive predictor of avoidance of patient in all 3 job categories. These findings were in line with our expectations and signal the need to reduce fear as a practical goal. Older age was a significant positive predictor for the concept of effectiveness in 2 job categories (nurses and others). Among nurses, older age was also a significant negative predictor for avoidance of patient and a significant positive predictor for acceptance of risk. Age has previously been shown to be a positive predictor for practicing SARS preventive measures among the general population (7). Hence, older age seems to correlate with an increased ability to cope with emergency situations related to infectious diseases. Type of facility (university hospital) was a significant negative predictor for the concept of effectiveness in 2 job categories (nurses and others). Although confined to the 7 facilities studied, university hospitals may have been less stringent in the formulating or implementing infection control measures, which in turn affected the overall concept of effectiveness of measures among healthcare workers.

### Limitations

Our study has several limitations. First, the cross-sectional nature of the study prevents assertion of cause and effect. Our conclusion, particularly on interrelationship among individual factors, is based on inferences. Second, responder bias may have been in play, i.e., only workers with a strong interest in SARS may have been motivated to respond, although the fairly high response rate counteracts this argument to an extent. Third, K-score may not accurately reflect knowledge of preventive measures. For example, workers who, in practice, had accurate knowledge about shoe covers as personal protective equipment may have answered incorrectly because they had been taught conflicting information. In fact, the Infectious Disease Surveillance Center (IDSC), Japan, categorizes shoe cover as an optional personal protective equipment (22). However, among the personal protective equipment considered in this study, only alcohol rubs (WHO) (2) and shoe cover (IDSC, Japan) (22) are considered optional, and the effect of conflicting information should not be strong. Fourth, we considered the difference in type of facility (university or nonuniversity) but did not consider differences by facility (hospital A or B) or type of unit (internal medicine, surgery, and others), which may be related to differences in job descriptions (even within the same job category) as well as the study variables. Such effects caused by affiliation constitute a separate theme worth further investigation, which will be pursued.

We found that the level of anxiety among healthcare workers in Japan was relatively high and that the implementation of preventive measures at the institutional level was not perceived to be sufficient. However, a collective assertion of 3 specific institutional measures stood out as the most important predictor for individual perception of risk, including avoidance of patient and acceptance of risk, as well as concept of general effectiveness of institutional measures. In view of the potential for future epidemics of SARS or other emerging infectious diseases, the planning and implementation of institutional measures should be given a high priority.

## Appendix

Excerpt from questionnaire.

### Knowledge of Preventive Measures

**"Do you believe the following measures are useful in protecting you from contracting SARS?"**

1. Area isolation
2. Hand washing
3. Alcohol rubs
4. Prominent notices
5. N95 mask
6. Gloves
7. Gowns
8. Surgical mask
9. Temperature checks
10. Hair cover
11. Paper mask
12. Goggles
13. Gauze mask
14. Shoe cover
15. Limiting visitors

**Concept of Institutional Measures**

1. "Were clear policies and protocols instituted for everyone to follow?" (clear policies and protocols)
2. "Do you have someone to turn to when you have a problem using the personal protective equipment?" (specialist available)
3. "Was there adequate training provided to you in the use of masks?" (adequate training)
4. "Do you feel that the implementations of protective measures at work are generally effective?" (effectiveness)

**Perception of Risk**

1. "Do you feel that you shouldn't be looking after patients with SARS?" (avoidance of patient)
2. "Do you accept the risk of getting SARS as part of your job?" (acceptance of risk)
3. "Do you have little control over whether you get infected or not?" (little personal control)
4. "Are you afraid of falling ill with SARS?" (fear)
5. "Are you looking for another job or considering resigning because of the risk?" (job change)

## Supplementary Material

Download PDF of Table A1 Knowledge of preventive measures, conception of institutional measures, and perception of risk by job category, sex, age, and type of facility.

## Appendix

**Table A1:** Knowledge of preventive measures, conception of institutional measures, and perception of risk by job category, sex, age, and type of facility. Download PDF of TA1 (223 KB, 1 page)

	Job category							Sex					Age					Type of facility					Total	
	Physicians		Nurses		Other			Men		Women			< 35 years		>35 years			University		Non-university*				
	(N = 1,370)		(N = 3,274)		(N = 2,638)			(N = 2,205)		(N = 5,077)			(N = 3,963)		(N = 3,319)			(N = 5,163)		(N = 2,119)			(N = 7,282)	
	n/N†	%	n/N	%	n/N	%	p value	n/N	%	n/N	%	p value	n/N	%	n/N	%	p value	n/N	%	n/N	%	p value	n/N	%
Knowledge of preventive measures																								
1. Area isolation	1,284/1,338	96.0	3,167/3,188	99.3	2,459/2,515	97.8	< 0.001	2,057/2,136	96.3	4,853/4,905	98.9	< 0.001	3,782/3856	98.1	3,128/3,185	98.2	0.724	4,876/4,985	97.8	2,034/2,056	98.9	< 0.001	6,910/7,041	98.1
2. Hand washing	1,279/1,338	95.6	3,196/3,222	99.2	2,510/2,566	97.8	< 0.001	2,064/2,150	96.0	4,921/4,976	98.9	< 0.001	3,826/3899	98.1	3,159/3,227	97.9	0.495	4,936/5,049	97.8	2,049/2,077	98.7	0.015	6,985/7,126	98.0
3. Alcohol rubs	1,149/1,320	87.0	2,980/3,150	94.6	2,364/2,487	95.1	< 0.001	1,900/2,116	89.8	4,593/4,841	94.9	< 0.001	3,566/3827	93.2	2,927/3,130	93.5	0.595	4,590/4,923	93.2	1,903/2,034	93.6	0.673	6,493/6,957	93.3
4. Prominent notices	1,154/1,330	86.8	2,911/3,191	91.2	2,245/2,496	89.9	< 0.001	1,829/2,124	86.1	4,481/4,893	91.6	< 0.001	3,465/3862	89.7	2,845/3,155	90.2	0.550	4,443/4,965	89.5	1,867/2,052	91	0.061	6,310/7,017	89.9
5. N95 mask	1,103/1,280	86.2	2,783/3,109	89.5	1,884/2,247	83.8	< 0.001	1,719/2,007	85.7	4,051/4,629	87.5	0.039	3,209/3712	86.4	2,561/2,924	87.6	0.175	3,999/4,670	85.6	1,771/1,966	90.1	< 0.001	5,770/6,636	86.9
6. Gloves	977/1,325	73.7	2,608/3,159	82.6	1,884/2,410	78.2	< 0.001	1,554/2,077	74.8	3,915/4,817	81.3	< 0.001	2,980/3820	78.0	2,489/3,074	81.0	0.003	3,763/4,871	77.3	1,706/2,023	84.3	< 0.001	5,469/6,894	79.3
7. Gowns	836/1,317	63.5	2,327/3,124	74.5	1,360/2,313	58.8	< 0.001	1,266/2,043	62.0	3,257/4,711	69.1	< 0.001	2,485/3778	65.8	2,038/2,976	68.5	0.019	3,041/4,761	63.9	1,482/1,993	74.4	< 0.001	4,523/6,754	67.0
8. Surgical mask	837/1,297	64.5	1,908/3,055	62.5	1,505/2,266	66.4	0.011	1,272/2,000	63.6	2,978/4,618	64.5	0.503	2,420/3749	64.6	1,830/2,869	63.8	0.535	2,996/4,693	63.8	1,254/1,925	65.1	0.323	4,250/6,618	64.2
9. Temperature checks	676/1,320	51.2	1,918/3,119	61.5	1,612/2,464	65.4	< 0.001	1,222/2,104	58.1	2,984/4,799	62.2	0.001	2,344/3803	61.6	1,862/3,100	60.1	0.189	2,966/4,900	60.5	1,240/2,003	61.9	0.289	4,206/6,903	60.9
10. Hair cover	726/1,318	55.1	1,982/3,103	63.9	1,315/2,323	56.6	< 0.001	1,155/2,053	56.3	2,868/4,691	61.1	< 0.001	2,141/3760	56.9	1,882/2,984	63.1	< 0.001	2,668/4,760	56.1	1,355/1,984	68.3	< 0.001	4,023/6,744	59.7
11. Paper mask	834/1,297	64.3	1,904/3,057	62.3	1,173/2,273	51.6	< 0.001	1,230/2,001	61.5	2,681/4,626	58.0	0.008	2,147/3771	56.9	1,764/2,856	61.8	< 0.001	2,793/4,696	59.5	1,118/1,931	57.9	0.237	3,911/6,627	59.0
12. Goggles	753/1,304	57.7	1,724/3,062	56.3	1,254/2,268	55.3	0.361	1,145/2,019	56.7	2,586/4,615	56.0	0.628	1,925/3706	51.9	1,806/2,928	61.7	< 0.001	2,476/4,679	52.9	1,255/1,955	64.2	< 0.001	3,731/6,634	56.2
13. Gauze mask	761/1,301	58.5	1,794/3,064	58.6	1,079/2,309	46.7	< 0.001	1,104/2,028	54.4	2,530/4,646	54.5	1.000	1,952/3787	51.5	1,682/2,887	58.3	< 0.001	2,567/4,726	54.3	1,067/1,948	54.8	0.746	3,634/6,674	54.5
14. Shoe cover	665/1,313	50.6	1,690/3,043	55.5	1,188/2,291	51.9	0.003	1,034/2,038	50.7	2,509/4,609	54.4	0.006	1,883/3711	50.7	1,660/2,936	56.5	< 0.001	2,317/4,690	49.4	1,226/1,957	62.6	< 0.001	3,543/6,647	53.3
15. Limiting visitors	398/1,333	29.9	1,274/3,086	41.3	727/2,383	30.5	< 0.001	663/2,101	31.6	1,736/4,701	36.9	< 0.001	1,268/3793	33.4	1,131/3,009	37.6	< 0.001	1,568/4,817	32.6	831/1,985	41.9	< 0.001	2,399/6,802	35.3
Knowledge (K-) score: sum of 15 items																								
11–15 (high)	684/1370	49.9	1876/3274	57.3	1150/2638	43.6	< 0.001	1,052/2,205	47.7	2,658/5,077	52.4	< 0.001	1,991/3,963	50.2	1,719/3,319	51.8	< 0.001	2,453/5163	47.5	1,257/2,119	59.3	< 0.001	3,710/7,282	50.9
6–10 (middle)	535/1370	39.1	1195/3274	36.5	1127/2638	42.7		876/2,205	39.7	1,981/5,077	39.0		1,647/3,963	41.6	1,210/3,319	36.5		2,157/5163	41.8	700/2,119	33.0		2,857/7,282	39.2
0–5 (low)	151/1370	11.0	203/3274	6.2	361/2638	13.7		277/2,205	12.6	438/5,077	8.6		325/3,963	8.2	390/3,319	11.8		553/5163	10.7	162/2,119	7.6		715/7,282	9.8
(mean ± SD)	(9.80 ± 3.30)		(10.44 ± 2.88)		(9.31 ± 3.32)		< 0.001	(9.62 ± 3.36)		(10.03 ± 3.07)		< 0.001	(9.94 ± 2.98)		(9.87 ± 3.37)		0.358	(9.68 ± 3.18)		(10.46 ± 3.07)		< 0.001	(9.91 ± 3.17)	
Concept of institutional measures																								
1. Clear policies and protocols	811/1292	62.8	2069/2930	70.6	1276/2148	59.4	< 0.001	1,261/2,013	62.6	2,895/4,357	66.4	0.003	2,171/3,524	61.6	1,985/2,846	69.7	< 0.001	2,829/4507	62.8	1,327/1,863	71.2	< 0.001	4,156/6,370	65.2
2. Specialist available	555/1302	42.6	1750/2934	59.6	1108/2200	50.4	< 0.001	912/2,007	45.4	2,501/4,429	56.5	< 0.001	1,749/3,582	48.8	1,664/2,854	58.3	< 0.001	2,232/4556	49.0	1,181/1,880	62.8	< 0.001	3,413/6,436	53.0
3. Adequate training	379/1288	29.4	1386/2836	48.9	691/2165	31.9	< 0.001	629/1,982	31.7	1,827/4,307	42.4	< 0.001	1,233/3,499	35.2	1,223/2,790	43.8	< 0.001	1,590/4449	35.7	866/1,840	47.1	< 0.001	2,456/6,289	39.1
4. Effectiveness	357/1314	27.2	1045/3076	34.0	715/2424	29.5	< 0.001	637/2,084	30.6	1,480/4,730	31.7	0.570	1,054/3,746	28.1	1,063/3,068	34.6	< 0.001	1,369/4822	28.4	748/1,992	37.6	< 0.001	2,117/6,814	31.1
Institutional (I-) score: sum of above measures 1–3.																								
3	211/1370	15.4	822/3274	25.1	362/2638	13.7	< 0.001	357/2,205	16.2	1,038/5,077	20.4	< 0.001	638/3,963	16.1	757/3,319	22.8	< 0.001	819/5163	15.9	576/2,119	27.2	< 0.001	1,395/7,282	19.2
2	249/1370	18.2	746/3274	22.8	458/2638	17.4		393/2,205	17.8	1,060/5,077	20.9		787/3,963	19.9	666/3,319	20.1		1,024/5163	19.8	429/2,119	20.2		1,453/7,282	20.0
1	374/1370	27.3	818/3274	25.0	722/2638	27.4		597/2,205	27.1	1,317/5,077	25.9		1,100/3,963	27.8	814/3,319	24.5		1,402/5163	27.2	512/2,119	24.2		1,914/7,282	26.3
0	536/1370	39.1	888/3274	27.1	1096/2638	34.6		858/2,205	38.9	1,662/5,077	32.7		1,438/3,963	36.3	1,082/3,319	32.6		1,918/5163	36.3	602/2,119	28.4		2,520/7,282	34.6
(mean ± SD)	(1.10 ± 1.09)		(1.46 ± 1.14)		(1.03 ± 1.07)		< 0.001	(1.11 ± 1.10)		(1.29 ± 1.13)		< 0.001	(1.16 ± 1.09)		(1.33 ± 1.15)		< 0.001	(1.14 ± 1.09)		(1.46 ± 1.17)		< 0.001	(1.24 ± 1.12)	
Perception of risk																								
1. Avoidance of patient	1149/1322	86.9	2982/3193	93.4	2327/2526	92.1	< 0.001	1,855/2,126	87.3	4,603/4,915	93.7	< 0.001	3,586/3,850	93.1	2,872/3,191	90.0	< 0.001	4,539/4984	91.1	1,919/2,057	93.3	0.002	6,458/7,041	91.7
2. Acceptance of risk	929/1337	69.5	2077/3209	64.7	1536/2522	60.9	< 0.001	1,431/2,136	67.0	3,111/4,932	63.1	0.002	2,414/3,873	62.3	2,128/3,195	66.6	< 0.001	3,207/5008	64.0	1,335/2,060	64.8	0.548	4,542/7,068	64.3
3. Little personal control	787/1317	59.8	1941/3146	61.7	1492/2495	59.8	0.267	1,246/2,106	59.2	2,974/4,852	61.3	0.098	2,304/3,802	60.6	1,916/3,156	60.7	0.941	2,980/4927	60.5	1,240/2,031	61.1	0.666	4,220/6,958	60.6
4. Fear	658/1345	48.9	1966/3245	60.6	1348/2588	52.1	< 0.001	1,054/2,165	48.7	2,918/5,013	58.2	< 0.001	2,191/3,924	55.8	1,781/3,254	54.7	0.352	2,654/5082	52.2	1,318/2,096	62.9	< 0.001	3,972/7,178	55.3
5. Job change	191/1336	14.3	1069/3132	34.1	608/2460	24.7	< 0.001	334/2,121	15.7	1,534/4,807	31.9	< 0.001	1,164/3,793	30.7	704/3,135	22.5	< 0.001	1,184/4906	24.1	684/2,022	33.8	< 0.001	1,688/6,928	27.0

*Nonuniversity includes municipal hospitals (2 facilities) and private hospitals (one facility). †n/N, number of respondents positively answering to questions about knowledge of preventive measures, concept of institutional measures, and perception of risk per number of respondents answering question (except for knowledge of paper mask and gauze mask, where negative answers were counted). Positive answer includes "probably agree," "agree," and "strongly agree", and negative answer includes "probably disagree," "disagree," and "strongly disagree." p value based on chi-square test for difference in proportion, t test for difference in 2 means, and ANOVA for differences in 3 means.
