# Supplementary material for: SARS Risk Perceptions in Healthcare Workers, Japan
Source: Emerg Infect Dis. 2005 Mar;11(3):404–10. doi: 10.3201/eid1103.040631 (PMC3298234; doi:10.3201/eid1103.040631)
Supplement: Download PDF of Table A1 — Knowledge of preventive measures, conception of institutional measures, and perception of risk by job category, sex, age, and type of facility. [file 04-0631-TA1.pdf]

Imai T, Takahashi K, Hoshuyama T, Hasegawa N, Lim M-K, Koh D. Perceptions of risk for SARS and countermeasures among healthcare workers, Japan. Emerg Infect Dis. 2005 Mar [*date cited*]. Available from http://www.cdc.gov/ncidod/EID/vol11no03/04-0631.htm

Appendix Table 2. Knowledge of preventive measures, conception of institutional measures, and perception of risk by job category, sex, age, and type of facility

	Job category							Sex					Age					Type of facility					Total	
	Physicians (N = 1,370)		Nurses (N = 3,274)		Other (N = 2,638)		p value	Men (N = 2,205)		Women (N = 5,077)		p value	< 35 years (N = 3,963)		≥35 years (N = 3,319)		p value	University (N = 5,163)		Non-university* (N = 2,119)		p value	(N = 7,282)	
	n/N†	%	n/N	%	n/N	%		n/N	%	n/N	%		n/N	%	n/N	%		n/N	%	n/N	%		n/N	%
Knowledge of preventive measures																								
1. Area isolation	1,284/1,338	96.0	3,167/3,188	99.3	2,459/2,515	97.8	< 0.001	2,057/2,136	96.3	4,853/4,905	98.9	< 0.001	3,782/3856	98.1	3,128/3,185	98.2	0.724	4,876/4,985	97.8	2,034/2,056	98.9	< 0.001	6,910/7,041	98.1
2. Hand washing	1,279/1,338	95.6	3,196/3,222	99.2	2,510/2,566	97.8	< 0.001	2,064/2,150	96.0	4,921/4,976	98.9	< 0.001	3,826/3899	98.1	3,159/3,227	97.9	0.495	4,936/5,049	97.8	2,049/2,077	98.7	0.015	6,985/7,126	98.0
3. Alcohol rubs	1,149/1,320	87.0	2,980/3,150	94.6	2,364/2,487	95.1	< 0.001	1,900/2,116	89.8	4,593/4,841	94.9	< 0.001	3,566/3827	93.2	2,927/3,130	93.5	0.595	4,590/4,923	93.2	1,903/2,034	93.6	0.673	6,493/6,957	93.3
4. Prominent notices	1,154/1,330	86.8	2,911/3,191	91.2	2,245/2,496	89.9	< 0.001	1,829/2,124	86.1	4,481/4,893	91.6	< 0.001	3,465/3862	89.7	2,845/3,155	90.2	0.550	4,443/4,965	89.5	1,867/2,052	91	0.061	6,310/7,017	89.9
5. N95 mask	1,103/1,280	86.2	2,783/3,109	89.5	1,884/2,247	83.8	< 0.001	1,719/2,007	85.7	4,051/4,629	87.5	0.039	3,209/3712	86.4	2,561/2,924	87.6	0.175	3,999/4,670	85.6	1,771/1,966	90.1	< 0.001	5,770/6,636	86.9
6. Gloves	977/1,325	73.7	2,608/3,159	82.6	1,884/2,410	78.2	< 0.001	1,554/2,077	74.8	3,915/4,817	81.3	< 0.001	2,980/3820	78.0	2,489/3,074	81.0	0.003	3,763/4,871	77.3	1,706/2,023	84.3	< 0.001	5,469/6,894	79.3
7. Gowns	836/1,317	63.5	2,327/3,124	74.5	1,360/2,313	58.8	< 0.001	1,266/2,043	62.0	3,257/4,711	69.1	< 0.001	2,485/3778	65.8	2,038/2,976	68.5	0.019	3,041/4,761	63.9	1,482/1,993	74.4	< 0.001	4,523/6,754	67.0
8. Surgical mask	837/1,297	64.5	1,908/3,055	62.5	1,505/2,266	66.4	0.011	1,272/2,000	63.6	2,978/4,618	64.5	0.503	2,420/3749	64.6	1,830/2,869	63.8	0.535	2,996/4,693	63.8	1,254/1,925	65.1	0.323	4,250/6,618	64.2
9. Temperature checks	676/1,320	51.2	1,918/3,119	61.5	1,612/2,464	65.4	< 0.001	1,222/2,104	58.1	2,984/4,799	62.2	0.001	2,344/3803	61.6	1,862/3,100	60.1	0.189	2,966/4,900	60.5	1,240/2,003	61.9	0.289	4,206/6,903	60.9
10. Hair cover	726/1,318	55.1	1,982/3,103	63.9	1,315/2,323	56.6	< 0.001	1,155/2,053	56.3	2,868/4,691	61.1	< 0.001	2,141/3760	56.9	1,882/2,984	63.1	< 0.001	2,668/4,760	56.1	1,355/1,984	68.3	< 0.001	4,023/6,744	59.7
11. Paper mask	834/1,297	64.3	1,904/3,057	62.3	1,173/2,273	51.6	< 0.001	1,230/2,001	61.5	2,681/4,626	58.0	0.008	2,147/3771	56.9	1,764/2,856	61.8	< 0.001	2,793/4,696	59.5	1,118/1,931	57.9	0.237	3,911/6,627	59.0
12. Goggles	753/1,304	57.7	1,724/3,062	56.3	1,254/2,268	55.3	0.361	1,145/2,019	56.7	2,586/4,615	56.0	0.628	1,925/3706	51.9	1,806/2,928	61.7	< 0.001	2,476/4,679	52.9	1,255/1,955	64.2	< 0.001	3,731/6,634	56.2
13. Gauze mask	761/1,301	58.5	1,794/3,064	58.6	1,079/2,309	46.7	< 0.001	1,104/2,028	54.4	2,530/4,646	54.5	1.000	1,952/3787	51.5	1,682/2,887	58.3	< 0.001	2,567/4,726	54.3	1,067/1,948	54.8	0.746	3,634/6,674	54.5
14. Shoe cover	665/1,313	50.6	1,690/3,043	55.5	1,188/2,291	51.9	0.003	1,034/2,038	50.7	2,509/4,609	54.4	0.006	1,883/3711	50.7	1,660/2,936	56.5	< 0.001	2,317/4,690	49.4	1,226/1,957	62.6	< 0.001	3,543/6,647	53.3
15. Limiting visitors	398/1,333	29.9	1,274/3,086	41.3	727/2,383	30.5	< 0.001	663/2,101	31.6	1,736/4,701	36.9	< 0.001	1,268/3793	33.4	1,131/3,009	37.6	< 0.001	1,568/4,817	32.6	831/1,985	41.9	< 0.001	2,399/6,802	35.3
Knowledge (K-) score: sum of 15 items																								
11–15 (high)	684/1370	49.9	1876/3274	57.3	1150/2638	43.6		1,052/2,205	47.7	2,658/5,077	52.4		1,991/3,963	50.2	1,719/3,319	51.8		2,453/5163	47.5	1,257/2,119	59.3		3,710/7,282	50.9
6–10 (middle)	535/1370	39.1	1195/3274	36.5	1127/2638	42.7	< 0.001	876/2,205	39.7	1,981/5,077	39.0	< 0.001	1,647/3,963	41.6	1,210/3,319	36.5	< 0.001	2,157/5163	41.8	700/2,119	33.0	< 0.001	2,857/7,282	39.2
0–5 (low)	151/1370	11.0	203/3274	6.2	361/2638	13.7		277/2,205	12.6	438/5,077	8.6		325/3,963	8.2	390/3,319	11.8		553/5163	10.7	162/2,119	7.6		715/7,282	9.8
(mean ± SD)	(9.80 ± 3.30)		(10.44 ± 2.88)		(9.31 ± 3.32)		< 0.001	(9.62 ± 3.36)		(10.03 ± 3.07)		< 0.001	(9.94 ± 2.98)		(9.87 ± 3.37)		0.358	(9.68 ± 3.18)		(10.46 ± 3.07)		< 0.001	(9.91 ± 3.17)	
Concept of institutional measures																								
1. Clear policies and protocols	811/1292	62.8	2069/2930	70.6	1276/2148	59.4	< 0.001	1,261/2,013	62.6	2,895/4,357	66.4	0.003	2,171/3,524	61.6	1,985/2,846	69.7	< 0.001	2,829/4507	62.8	1,327/1,863	71.2	< 0.001	4,156/6,370	65.2
2. Specialist available	555/1302	42.6	1750/2934	59.6	1108/2200	50.4	< 0.001	912/2,007	45.4	2,501/4,429	56.5	< 0.001	1,749/3,582	48.8	1,664/2,854	58.3	< 0.001	2,232/4556	49.0	1,181/1,880	62.8	< 0.001	3,413/6,436	53.0
3. Adequate training	379/1288	29.4	1386/2836	48.9	691/2165	31.9	< 0.001	629/1,982	31.7	1,827/4,307	42.4	< 0.001	1,233/3,499	35.2	1,223/2,790	43.8	< 0.001	1,590/4449	35.7	866/1,840	47.1	< 0.001	2,456/6,289	39.1
4. Effectiveness	357/1314	27.2	1045/3076	34.0	715/2424	29.5	< 0.001	637/2,084	30.6	1,480/4,730	31.7	0.570	1,054/3,746	28.1	1,063/3,068	34.6	< 0.001	1,369/4822	28.4	748/1,992	37.6	< 0.001	2,117/6,814	31.1
Institutional (I-) score: sum of above measures 1–3.																				,				
3	211/1370	15.4	822/3274	25.1	362/2638	13.7		357/2,205	16.2	1,038/5,077	20.4		638/3,963	16.1	757/3,319	22.8		819/5163	15.9	576/2,119	27.2		1,395/7,282	19.2
2	249/1370	18.2	746/3274	22.8	458/2638	17.4	< 0.001	393/2,205	17.8	1,060/5,077	20.9	< 0.001	787/3,963	19.9	666/3,319	20.1	< 0.001	1,024/5163	19.8	429/2,119	20.2	< 0.001	1,453/7,282	20.0
1	374/1370	27.3	818/3274	25.0	722/2638	27.4		597/2,205	27.1	1,317/5,077	25.9		1,100/3,963	27.8	814/3,319	24.5		1,402/5163	27.2	512/2,119	24.2		1,914/7,282	26.3
0	536/1370	39.1	888/3274	27.1	1096/2638	34.6		858/2,205	38.9	1,662/5,077	32.7		1,438/3,963	36.3	1,082/3,319	32.6		1,918/5163	36.3	602/2,119	28.4		2,520/7,282	34.6
(mean ± SD)	(1.10 ± 1.09)		(1.46 ± 1.14)		(1.03 ± 1.07)		< 0.001	(1.11 ± 1.10)		(1.29 ± 1.13)		< 0.001	(1.16 ± 1.09)		(1.33 ± 1.15)		< 0.001	(1.14 ± 1.09)		(1.46 ± 1.17)		< 0.001	(1.24 ± 1.12)	
Perception of risk																								
1. Avoidance of patient	1149/1322	86.9	2982/3193	93.4	2327/2526	92.1	< 0.001	1,855/2,126	87.3	4,603/4,915	93.7	< 0.001	3,586/3,850	93.1	2,872/3,191	90.0	< 0.001	4,539/4984	91.1	1,919/2,057	93.3	0.002	6,458/7,041	91.7
2. Acceptance of risk	929/1337	69.5	2077/3209	64.7	1536/2522	60.9	< 0.001	1,431/2,136	67.0	3,111/4,932	63.1	0.002	2,414/3,873	62.3	2,128/3,195	66.6	< 0.001	3,207/5008	64.0	1,335/2,060	64.8	0.548	4,542/7,068	64.3
3. Little personal control	787/1317	59.8	1941/3146	61.7	1492/2495	59.8	0.267	1,246/2,106	59.2	2,974/4,852	61.3	0.098	2,304/3,802	60.6	1,916/3,156	60.7	0.941	2,980/4927	60.5	1,240/2,031	61.1	0.666	4,220/6,958	60.6
4. Fear	658/1345	48.9	1966/3245	60.6	1348/2588	52.1	< 0.001	1,054/2,165	48.7	2,918/5,013	58.2	< 0.001	2,191/3,924	55.8	1,781/3,254	54.7	0.352	2,654/5082	52.2	1,318/2,096	62.9	< 0.001	3,972/7,178	55.3
5. Job change	191/1336	14.3	1069/3132	34.1	608/2460	24.7	< 0.001	334/2,121	15.7	1,534/4,807	31.9	< 0.001	1,164/3,793	30.7	704/3,135	22.5	< 0.001	1,184/4906	24.1	684/2,022	33.8	< 0.001	1,688/6,928	27.0

*Nonuniversity includes municipal hospitals (2 facilities) and private hospitals (one facility).
†n/N, number of respondents positively answering to questions about knowledge of preventive measures, concept of institutional measures, and perception of risk per number of respondents answering question (except for knowledge of paper mask and gauze mask, where negative answers were counted). Positive answer includes "probably agree," "agree," and "strongly agree", and negative answer includes "probably disagree," "disagree," and "strongly disagree." p value based on chi-square test for difference in proportion, t test for difference in 2 means, and ANOVA for differences in 3 means.
